# Proof of concept study of the posterior quadratus lumborum block for laparoscopic myomectomy: A randomized controlled trial

**DOI:** 10.1371/journal.pone.0321890

**Published:** 2025-04-22

**Authors:** Christopher Little, Natale Naim, Tristan Grogan, Steve Yu, Pamela A. Chia

**Affiliations:** 1 Department of Anesthesiology and Perioperative Medicine, University of California, Los Angeles, Los Angeles, California, United States of America; 2 Department of Medicine Statistics Core, University of California, Los Angeles, Los Angeles, California, United States of America; 3 Minimally Invasive Gynecological Surgery, Hoag Hospital Newport Beach, Newport Beach, California, United States of America; University of Brescia Department of Clinical and Experimental Sciences: Universita degli Studi di Brescia Dipartimento di Scienze Cliniche e Sperimentali, ITALY

## Abstract

In the United States, 65,000 myomectomies are performed annually to treat uterine fibroids. The quadratus lumborum block (QLB) is an effective block for laparoscopic pelvic surgery, urologic surgery, hip surgery and cesarean sections, with limited data for laparoscopic myomectomies. We evaluated the posterior QLB in reducing MME consumption and numeric rating scale (NRS) pain scores in patients undergoing laparoscopic myomectomies. Twenty-two subjects were enrolled in this single-center, randomized, controlled study between March 28, 2019 and June 16, 2020 and were randomized to either the QLB or control group. Seven subjects were excluded from the final analysis, 5 after being lost to follow-op and 2 for not meeting the inclusion criteria. Recipients in the QLB group received bilateral posterior QLBs, with 30 mL of 0.25% ropivacaine per injection. The primary outcome of MME use at 24 hours was not significant between the QLB group and the control group (23.3 ± 8.5 mg vs. 25.7 ± 14.4 mg, p = 0.859). The secondary outcome of NRS pain scores was also not significant between groups (p > 0.05). While this study did not provide evidence that QLB may be useful in reducing opioid consumption or pain scores in patients undergoing laparoscopic myomectomies, further studies with a larger sample size will be valuable to determine the effectiveness of this block for laparoscopic myomectomy.

## Introduction

In the United States, 65,000 myomectomies are performed each year to treat uterine fibroids [1–3]. These patients could potentially benefit from regional anesthesia in an effort to minimize perioperative opioid use and its associated adverse effects such as nausea, pruritis, and respiration depression. Patients that elect for myomectomies are often young women who may be already at risk for post-operative nausea following laparoscopic surgery and may benefit from opioid-sparing techniques to reduce this risk. A recent systematic review and meta-analysis demonstrated that transversus abdominis plane (TAP) blocks are superior to wound infiltration with local anesthetic for pain management in gynecological surgery [4]. These findings highlights the potential for further research into the use of plane blocks to benefit this patient population.

The quadratus lumborum block (QLB) is an abdominal plane block, where its analgesic effect occurs through local anesthetic deposition covering the thoracolumbar fascia and possibly the thoracic paravertebral space [5–9]. QLBs have been used for cesarean sections [10–12], hip surgery [13], laparoscopic pelvic surgery [14], and urologic surgery [15]. A meta-analysis demonstrated the superiority of QLBs over systemic opioids for pain management in cesarean sections; however, there is limited evidence in the literature for the efficacy of QLBs in laparoscopic myomectomy specifically [10,16,17]. We propose the QLB could also be of benefit for laparoscopic myomectomies, and hypothesized that subjects who received a posterior QLB would have lower opioid use than subjects in the control group 24 hours after surgery.

## Methods

The Institutional Review Board (IRB) approved this study (IRB 18-000825), and this trial was registered with ClinicalTrials.gov on February 26, 2019 (NCT03935815). Between March 28, 2019 and June 16, 2020, 22 subjects were recruited and randomized to either the QLB or control group.

This trial was a single center, single-blinded, randomized, controlled study. Inclusion criteria were subjects aged 18-years or older, had an ASA physical status of 1 or 2, and were undergoing laparoscopic myomectomy. Exclusion criteria were those with a history of chronic pain on preoperative opioids, coagulopathy, localized infections, on anticoagulation, body mass index (BMI) greater than 35, or non-English speaking. We obtained written informed consent for study participation preoperatively. Randomization was performed by a third-party statistician via a random number generator, and treatment allocations were placed in sealed opaque sequenced envelopes. Subjects were blinded to the treatment they received. Those in the QLB group received bilateral posterior QLBs with 30 mL of 0.25% ropivacaine per injection, after induction of general anesthesia with endotracheal intubation. The acute pain service performed the blocks with a 2–5 MHz curvilinear or 5–10 MHz linear probe (rC60xi, L38xi, Sonosite SII, FUJIFILM-Sonosite, Tokyo, Japan) and a 21G 100 mm insulated needle (Stimuplex®, BBraun, Mesulngen Germany). Local anesthesia was injected by hydro-dissecting the lumbar interfascial triangle with an in-plane approach. A sham was not performed in the control group.

All subjects received 1000 mg PO acetaminophen preoperatively, and dexamethasone 10 mg IV, ondansetron 4 mg IV, and 15 mg IV ketorolac, intraoperatively. Postoperatively, hydromorphone 0.2 mg IV and oxycodone 5 mg PO for mild pain, hydromorphone 0.4 mg IV and oxycodone 10 mg PO for moderate pain, and fentanyl 25 mcg for severe pain were available. Subjects were allowed to use acetaminophen and ibuprofen upon discharge. Our primary outcome was morphine milligram equivalents (MME) at 24 hours following surgery. Our secondary outcome was numeric rating scale (NRS) pain scores up to 48 hours after the end of surgery. Pain scores were assessed upon arrival to PACU (t = 0), and at 1 h, 2 h, 3 h by the PACU nurse and at 6 h, 24 h and 48 h after surgery by the patient.

### Laparoscopic myomectomy surgical procedure

For the surgery, four trocars were placed. The primary trocar used was a 5 mm non-bladed trocar placed at the umbilicus using a direct optical entry approach. On the right quadrant two secondary trocars were placed. The most lateral trocar placed was a 12 mm non-bladed trocar. Medial to the 12 mm trocar, a 5 mm non-bladed trocar was placed. On the left quadrant one 5 mm non-bladed trocar was placed. All secondary trocars were placed between the level of the umbilicus and the level of the anterior superior iliac spine.

Dilute vasopressin was injected into the leiomyoma pseudo-capsule. Uterine incision was performed using a harmonic scalpel. The fibroid was grasped with a single tooth tenaculum and counter traction provided with a non-traumatic grasper. The pseudo-capsule was circumferentially dissected with a harmonic scalpel. After the leiomyoma removal, the uterus was repaired in multiple layers. All leiomyomas were removed by extracorporeal mechanical morcellation.

### Statistical analysis

Study outcomes (MME and NRS pain scores) were compared between groups at each time point using Wilcoxon rank-sum tests, as a sensitivity analysis, generalized estimating equations (GEE) models were also used to evaluate group differences over time for these outcomes. The GEE models yielded conclusions consistent with the primary analysis (results not shown). Analyses were carried out using IBM SPSS V25 (Armonk, NY) and *p*-values less than 0.05 were considered statistically significant. The statistical analysis was completed after the data was collected, by a statistician who was not involved with data collection.

## Results

**Fig 1:**
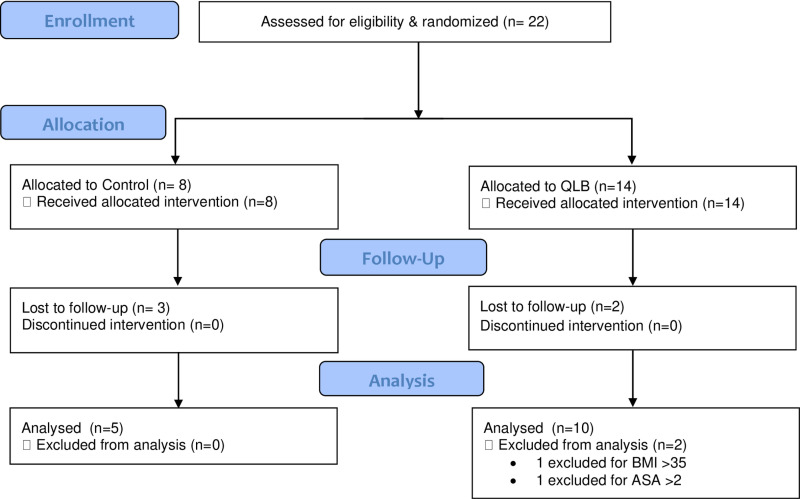
CONSORT diagram. Twenty two subjects were enrolled into this study and randomly assigned to the control or QL group. Seven subjects were excluded from the final data analysis, including five subjects who were lost to follow-up and two subjects who did not meet the inclusion criteria.

Of the 22 subjects recruited, 15 were included in the final data analysis after 5 subjects were lost to follow-op and 2 were excluded for not meeting the inclusion criteria (Fig 1). Table 1 summarizes the subject and surgery characteristics in the QLB and control groups. The primary outcome of MME consumption (mean ± standard deviation) at 24 hours was not significant between the QLB group and the control group (23.3 ± 8.5 mg vs. 25.7± 14.4 mg, p = 0.859) or at any other time point (Table 2). Mean NRS pain scores (mean ± standard deviation) were also not significantly different between groups at the time points measured up to 48 h (p > 0.05, Table 3). There were no serious adverse events reported in the QLB or control groups.

**Table 1:** Subject and surgery characteristics.

	QLB (n = 10)	Control (n = 5)
Age	36.4 ± 6.3	42.4 ±8.9
BMI (kg/m^2^)	23.6 ± 3.4	25.9 ± 4.4
ASA score		
ASA I	6 (60%)	1 (20%)
ASA II	4 (40%)	4 (80%)
Prior pelvic surgery	4 (40%)	1 (20%)
Average surgery time (h)	4.2 (0.9)	3.8 (1.1)
Estimated blood loss (EBL)		
Minimal (<50 mL)	3 (30%)	2 (40%)
Low (50–100 mL)	5 (50%)	2 (40%)
Moderate (101–200 mL)	2 (20%)	1 (20%)
Total fibroids removed^a^		
Fibroids removed (<5)	4 (40%)	3 (60%)
Fibroids removed (5–10)	2 (20%)	0 (0%)
Fibroids removed (>10)	4 (40%)	2 (40%)

Results are presented as mean ± SD for age and BMI, and as a number and percentage of subjects for ASA I and II status, prior pelvic surgery, EBL, and total number of fibroids removed.

^a^All fibroids removed ranged from Type 1–7.

ASA, American Society of Anesthesiologists; BMI, body mass index; QLB, quadratus lumborum block; EBL, estimated blood loss.

**Table 2:** Morphine milligram equivalent (MME) consumption.

Time	QLB (n = 10) (mean ± SD)	Control (n = 5) (mean ± SD)	*p*-value
Intraoperative MME	14.5 ± 7.9	13.3 ± 5.4	0.953
PACU MME	5.1 ± 2.3	8.8 ± 10	1
MME at 6 hrs	1.5 ± 1.7	1.2 ± 0.7	1
MME 7–24 hrs	2.3 ± 1.7	2.4 ± 3.3	0.859
MME 25–48 hrs	2.3 ± 2.2	2.1 ± 2.0	1
MME total at 24 hrs	23.3 ± 8.5	25.7 ± 14.4	0.859

The above values are represented as mean ± SD.

QLB, quadratus lumborum block; MME, morphine milligram equivalents; PACU, Post-Anesthesia Care Unit.

**Table 3:** Post-operative numeric rating scale (NRS) pain scores.

Time (hours)	QLB (n = 10) (mean ± SD)	Control (n = 5) (mean ± SD)	*p*-value
0	6.8 ± 3.2	6.8 ± 1.9	0.859
1	4.1 ± 3.0	5.2 ± 2.9	0.594
2	4.7 ± 3.0	5.0 ± 2.4	0.859
3	3.8 ± 2.7	5.0 ± 1.7	0.371
6	2.1 ± 2.3	5.2 ± 2.4	0.055
24	4.6 ± 2.4	5.4 ± 1.5	0.438
48	5.1 ± 1.8	4.0 ± 0.8	0.260

The above values are represented as mean ± SD.

NRS, numeric rating scale; QLB, quadratus lumborum block.

## Discussion

The goal of this study was to assess the utility of the QLB for postoperative pain after laparoscopic myomectomy surgery. Our study found that the QLB did not reduce MME or pain scores compared to the control group. This was consistent with the mixed results among studies looking specifically at the efficacy of QLB for laparoscopic gynecological surgeries. One study demonstrated the QLB group had improved pain scores at 1 h, 3 h, and 24 h after surgery [16], while another study did not find any difference between the QLB compared to the control group [17]. Future research should aim to investigate the effects of the QLB on specific surgical procedures to better define its clinical utility. While our study was underpowered to make recommendations, this pilot study suggests the feasibility and potential benefit of a QLB in this patient population.

Studies have demonstrated the TAP block, another abdominal fascial plane block, may help reduce pain scores and opioid consumption [4], and the QLB has been proposed as another valuable regional technique, for its ability to potentially provide analgesia to larger area and manage visceral pain [12]. Inadequate pain control is a common reason for delays in discharge and unintended postoperative admissions [18–20]. As more operations are performed in the outpatient setting, prioritizing perioperative pain management is paramount for prompt discharge, and the use of regional techniques to improve postoperative pain and reduce opioid exposure is becoming more prevalent.

The main limitation of this study was the small sample size. Our sample size (QLB group n = 10 vs control group n = 5) was only powered to reliably detect differences as small as 1.66 between groups (>80% power, two sample t-test, alpha = 0.05). For our primary outcome, MME at 24 hours, we only observed an effect size of 0.21. Preliminary data warrants a follow up study to fully determine the impact of this block on MME consumption and postoperative analgesic effects following laparoscopic myomectomy. A second limitation was our inability to assess block success through dermatomal means because the block was placed after induction of general anesthesia and assessing the patients awake would have risked unblinding them. Lastly, we elected not to include a sham for the controls. QLBs for laparoscopic myomectomies were not currently the standard of care during the study, so we decided not expose subjects to potential block complications like bowel and renal injury and hematoma formation for ethical reasons.

A strength of this study was that a single surgeon performed all laparoscopic myomectomy procedures, ensuring standardized surgical technique. In addition, all subjects were subject to the same post operative multimodal analgesic plan, with the use of non-opioid adjuncts including acetaminophen, ketorolac, and ibuprofen. Future studies should control for these confounders to ascertain the analgesic effect of the QLB on MME values and NRS pain scores. Lastly, it is important to acknowledge that regional anesthesia is a key component of enhanced recovery after surgery protocols, which aim to reduce postoperative opioid use. The adoption of these protocols have been hindered by poor compliance and slow implementation [21]. Additional research highlighting the benefits of regional anesthesia in gynecological procedures, such as laparoscopic myomectomy, could help support its broader integration into these protocols.

## Conclusion

While this pilot study did not find evidence to demonstrate that the QLB is effective at reducing opioid consumption or NRS pain scores in patients receiving laparoscopic myomectomies, further studies with a larger sample size will be valuable to determine the effectiveness of this block in laparoscopic myomectomy.

## Supporting information

S1 File Study data. (XLSX)

S2 File CONSORT 2010 checklist 2. (DOCX)
